# Heterogeneity of BCR-ABL rearrangement in patients with chronic myeloid leukemia in Pakistan

**Published:** 2014

**Authors:** Najia Tabassum, Mohammad Saboor, Rubina Ghani, Moinuddin Moinuddin

**Affiliations:** 1 Najia Tabassum, M.Phil, Baqai Institute of Hematology, Baqai Medical University, Karachi, Pakistan.; 2 Mohammed Saboor, PhD, Baqai Institute of Hematology, Baqai Medical University, Karachi, Pakistan.; 3 Rubina Ghani. PhD, Department of Biochemistry, Baqai Medical University, Karachi, Pakistan.; 4 Moinuddin Moinuddin, FRCP (E), FRCP (C), Baqai Institute of Hematology, Baqai Medical University, Karachi, Pakistan.

**Keywords:** BCR-ABL, Chronic Myeloid Leukaemia, Ph chromosome

## Abstract

***Background and Objective:*** Breakpoint cluster region-Abelson (BCR-ABL) rearrangement or Philadelphia (Ph) chromosome in Chronic Myeloid Leukemia (CML) is derived from a reciprocal chromosomal translocation between ABL gene on chromosome 9 and BCR gene on chromosome 22. This chimeric protein has various sizes and therefore different clinical behaviour. The purpose of this study was to determine the heterogeneity of BCR-ABL rearrangement in patients with Ph^+^CML in Pakistan.

***Methods:*** The study was conducted at Civil Hospital and Baqai Institute of Hematology (BIH) Karachi. Blood samples from 25 patients with CML were collected. Multiplex reverse transcription polymerase chain reaction (RT-PCR) was performed to identify various BCR-ABL transcripts.

***Results:*** All 25 samples showed BCR-ABL rearrangements. Out of these, 24 (96%) patients expressed p210 BCR-ABL rearrangements i.e. 60% (n=15) had b3a2 and 32% (n=8) had b2a2 rearrangements. Co-expression of b3a2 /b2a2 rearrangement and p190 (e1a3) rearrangement was also identified in two patients.

***Conclusion:*** It is apparent that majority of the patients had p210 BCR-ABL rearrangements. Frequency of co-expression and rare fusion transcripts was very low.

## INTRODUCTION

CML contributes to 20% of all leukemias diagnosed in adults. It is said to be the first human malignancy that is linked to a single acquired genetic abnormality. Pathognomonic marker of CML is the Ph chromosome that results from a reciprocal chromosomal translocation between the long arms of chromosome 9 and 22. This fuses the ABL gene on chromosome 9 with the BCR gene on chromosome 22 producing an oncogene called BCR-ABL.^1^ Each fusion protein has a constant size of ABL protein but differ in the length of BCR protein.^2^ ABL has 11 exons and are denoted as “a”. Alternative splicing of the first exon result in two isoforms, 1a and 1b.^3^ Breakpoints in ABL gene are between exon 1a or 1b and a2.^4^ BCR has 23 exons and are denoted as “e”. Breaks in the BCR gene occurs in one of the following three regions i.e. Major (M), minor (m) and micro (μ) BCR. M-BCR extends from exon 12 to 16. Breakpoint in M-BCR joins exon 13 (e13) or exon 14 (e14) with exon 2 (a2) of ABL. This results in fusion transcripts e13a2 and e14a2 respectively translating into a 210kDa (p210^BCR-ABL^) protein. P210^BCR-ABL^ is detected in more than 95% of cases with Ph^+^CML and one third of cases with Ph^+^ Acute Lymphoblastic Leukemia (Ph^+ ^ALL).^5^ m-BCR involves intron 1 and joins exon 1 (e1) with a2 resulting in a smaller fusion transcript, e1a2. It codes for a 190kDa (p190^BCR-ABL^) protein.^4^ It is seen primarily in Ph^+^ALL and in rare cases of CML. Monocytosis is predominantly seen in this type of CML. Low level of expression of these p190-type transcripts compared to p210 indicates that these are most likely the result of alternative splicing of the primary mRNA.^6^ μ-BCR involves intron 19 and results in joining of exon 19(e19) of BCR with a2 of ABL and results in e19a2. It encodes a 230kDa (p230^BCR-ABL^) protein. This rare fusion is seen in neutrophilic CML (N-CML) and sometimes in Ph^+ ^CML and Acute Myeloid Leukemia (AML).^4^^,^^6^

All BCR-ABL fusion proteins display activated tyrosine kinase activity. P190^BCR-ABL^ has a higher activity than p210^BCR-ABL^ resulting in a greater potential to induce a malignant change.^1^ Occasional cases with other junctions such as b2a3, b3a3, e1a3, e6a2 or e2a2 have been reported in patients with ALL and CML.^4^

## METHODS

This was an observational cross sectional study conducted at BIH and Civil hospital, Karachi.

***Inclusion criteria were:***

1. Newly diagnosed patients with CML or patients with CML treated with Hydroxyurea.
2. Presence of Ph chromosome or BCR-ABL rearrangement.
3. Age ≥18yrs of either sex.

***Exclusion criteria were:***

1. BCR-ABL negative CML
2. History of any myelo-poliferative disease (MPD) such as polycythemia vera (PV), essential thrombocythemia (ET) and idiopathic myelofibrosis (IMF)
3. Patients treated with tyrosine kinase inhibitor

A total of 25 patients with CML during the above mentioned period and fulfilling the inclusion criteria were enrolled in this study. Written informed consent was taken. This study was approved by ethical committee of BMU. Data were recorded on case report forms. Age, gender, first and last complete blood counts (CBC), bone marrow biopsy reports were recorded. Whole blood samples (10cc each) were collected in EDTA (Ethylene diamine tetra acetic acid) tubes. For identification of break points in BCR-ABL, extracted RNA from plasma was used. RT-PCR was performed according to Seeplex kit method. Cycling conditions are mentioned in Table-I. Test bands along with positive control were visualized using gel transilluminator. Sizes of the PCR products were determined by comparing them with a DNA ladder. Gel was interpreted according to the BCR-ABL marker (M) using Seeplex leukemia detection user manual as a reference. M was used to amplify the approximate size of target product run on a gel electrophoresis. BCR-ABL positive control (PC) was a mixture of BCR-ABL (b2a2, e1a2). Both M and PC were present in the kit.

***Statistical analysis:*** Statistical package for social sciences (SPSS) version 16 was used for data analysis. Descriptive statistics was applied for calculating the frequency.

## RESULTS

**Fig.1:**
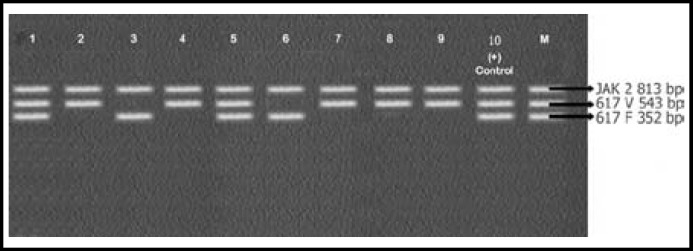
caption

**Fig.2:**
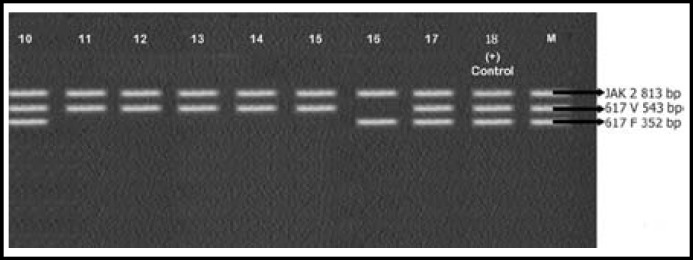
caption

**Fig.3:**
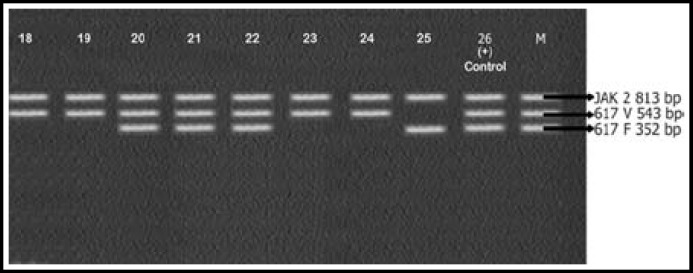
caption

Mean age of the patients enrolled in this study was 51±2.5 years. CBC of patients showed increased leukocyte count with complete left shift. Bone marrow findings were consistent with that of CML. All 25 samples were positive for BCR-ABL rearrangements. Results are shown in Fig. 1, 2, 3 and Table-II. Most patients 24/25 (96%) expressed p210 BCR-ABL rearrangement. Out of these 24 cases, 15 (60%) showed b3a2 while 8 (32%) cases had b2a2 rearrangement. There was only 1 (4%) case of co-expression of b3a2/b2a2 rearrangement. One case of p190 BCR-ABL rearrangement e1a3 (4%) was also identified.

## DISCUSSION

Different types of BCR-ABL rearrangement are associated with different clinical course and prognosis. Incidence of CML and frequency of various BCR-ABL transcripts differs among different ethnic backgrounds.^7^

Several molecular techniques like Southern blot, FISH (should be written in full) and Conventional RT-PCR are currently used for the detection of BCR-ABL gene. However, Conventional RT-PCR without cytogenetics can miss the detection of rare cases if proper primers are not used. Multiplex RT-PCR is similar to Conventional RT-PCR but includes more than one pair of primers. Presently it is considered a reliable technique to identify typical and atypical BCR-ABL transcripts in a single reaction.^5^

**Table-I:** Cycling conditions of RT-PCR in BCR-ABL

***Segment***	***Number of cycles***	***Temperature***	***Duration***
Initial duration	01	940C	15 mins
Denaturation	37	940C	0.5 min
Annealing	37	600C	1.5 mins
Extension	37	720C	1.5 mins
Final extension	01	720C	10 mins

**Table-II:** Frequency of expression of BCR-ABL breakpoints in CML

***BCR-ABL breakpoints***	***% Frequency(n=25)***
b3a2	56%
b2a2	8%
b3a2/b2a2	4%
e1a3	4%
e1a2	4%

In his study Multiplex RT-PCR was used to analyze BCR-ABL rearrangements in patients with CML. Primers used in this study detected eight types of BCR-ABL rearrangements in one PCR reaction. Hence it was possible to detect typical BCR-ABL fusion transcript such as b2a2 and b3a2 and atypical types lacking ABL exon a2 such as b2a3 and b3a3. Transcripts resulting from BCR breakpoints outside M-BCR such as e1a2 and e1a3 were also analysed. Overall, 92% of the patient had fusions genes involving the M-BCR region corresponding to p210 protein. Out of these, 60% had b3a2 while 32% had b2a2 rearrangement. Therefore the number of patients with b3a2 was twice the number of patients with b2a2 as shown in Table-I. The above findings of BCR-ABL rearrangement in our study is similar to those reported by Iqbal et al.^8^ Their data on Pakistani population with Ph^+^CML showed the frequency of b3a2 and b2a2 to be 63.33% and 36.66% respectively. Similar frequencies in different populations have been reported earlier in literature. Reiter et al.^9^ found the incidence of b3a2 and b2a2 rearrangements in patients with CML 68.4% and 31.6% respectively. Verschreagen et al.^10^ found the frequency of b3a2 and b2a2 to be 67.9% and 30.2% respectively. Yaghmaie et al.^4^ reported that in Iranian population b3a2 was 63% and b2a2 was 20%. Goh et al.^5^ reported that in Korean population 67.6% had b3a2 while 32.34% had b2a2. Ito et al.^11^ reported the frequency of b3a2 transcript in Japanese population was 67.50% while 30.20% had b2a2. In Thailand, 61% had b3a2 while 31% had b2a2 as reported by Udomsakdi et al.^12^

In our study majority of the patients like other reported, expressed p210 BCR-ABL rearrangement. Expression of b3a2 and b2a2 rearrangements was in accordance with Korean, Japanese, Thai and Mexican populations. There was neither an expression of p230 nor was there any case of co-expression of p190/p210 BCR-ABL transcript.

Only one case (4%) expressed more than one type of mRNA i.e. b2a3/ b2a2 transcript. Co-expression could be due to alternative splicing or phenotypic variation. Rarely may it be due to existence of several leukemic cell lines with different BCR-ABL transcript expression.^13^ Co-expression of two or more transcript has been reported by Yaghmaie et al.^4^, Goh et al.^5^ and Henegariu et al.^13^ but its incidence is rare.

One case of rare p190 transcript e1a3 was identified in our study. This is the third case of e1a3 transcript in a patient with Ph ^+^CML. First case was identified by Roman et al.^14^ and the second was reported by Goh et al.^5^ using Multiplex RT PCR. Till date no further case has been reported. Reports suggest that P190^BCR-ABL^CML is associated with an inferior outcome to Tyrosine kinase inhibitor therapy.^15^

Relatively few studies are available regarding the significance of BCR-ABL transcript type. Some reports suggest that type of transcripts may have clinical significance. They may help in the understanding pathobiology of t (9; 22)-positive leukemic cells. Perego et al.^16^ reported that CML patients with b3a2 transcripts had higher platelet count than those with b2a2. Prejzner et al.^17^ suggested that patients with b3a2 transcripts had longer survival than those with b2a2.

Therefore it is recommended that a comprehensive survey on Pakistani patients with CML should be carried out and all patients must be followed to study the clinical behaviour and prognosis of each fusion transcript.
